# Conservative Management of Aberrant Right Subclavian Artery (Arteria Lusoria): A Case Report and Literature Review

**DOI:** 10.7759/cureus.82608

**Published:** 2025-04-19

**Authors:** Yogesh Acharya, Omar Merican, Kevin Van der Merwe, Kalliopi-Maria Tasopoulou, Vikrant Parihar

**Affiliations:** 1 Gastroenterology, Letterkenny University Hospital, Letterkenny, IRL; 2 Vascular Surgery, Western Vascular Institute, Galway University Hospital, Galway, IRL

**Keywords:** aberrant right subclavian artery, arteria lusoria, case report, conservative treatment, kommerell's diverticulum

## Abstract

The most common embryological aortic arch abnormality is the aberrant right subclavian artery (ARSA), also known as arteria lusoria or dysphagia lusoria. Arteria lusoria is commonly associated with vascular anomalies, including truncus bicaroticus, right-sided aortic arch, and Kommerell's diverticulum (KD), predisposing to complications like acute aortic syndrome. Surgical intervention is usually reserved for symptomatic patients with compressive and/or hemodynamic alterations. However, there is no consensus on its clinical management and subsequent follow-up. Here, we present the case of a 71-year-old symptomatic elderly female with arteria lusoria who was managed conservatively. We believe conservative management is a viable option, especially in elderly patients, which should be supported with regular surveillance to detect any complications that necessitate surgical intervention.

## Introduction

Aberrant right subclavian artery (ARSA), also known as arteria lusoria or dysphagia lusoria, although uncommon (0.5% to 2%), is the most common embryological aortic arch abnormality [1,2]. It was first described by Hunauld in 1735 in animals and subsequently by Bayford as a clinical entity in a lady with dysphagia [3,4]. The arch of the aorta gives rise to three arteries: the brachiocephalic trunk (further divides into the right subclavian artery and the right common carotid artery), the left common carotid artery, and the left subclavian artery. In the case of the ARSA, the brachiocephalic trunk is typically absent [1]. Instead, four arteries arise from the arch, i.e., the right and left common carotid arteries, the left subclavian artery, and, most distally, the right subclavian artery, which crosses the midline and runs behind the oesophagus [1,2]. 

Arteria lusoria is commonly associated with vascular anomalies, including truncus bicaroticus, right-sided aortic arch, and Kommerell's diverticulum (KD), predisposing to complications like acute aortic syndrome [1]. However, there is no consensus on clinical management and follow-up. Here, we report the case of a 71-year-old female with arteria lusoria who was managed conservatively.

## Case presentation

A 71-year-old female presented to the gastroenterology clinic following a two-year history of dysphagia and a sensation of food sticking in the oesophagus. Her dysphagia was limited to solid foods, and symptoms were not exacerbated by positional change. She did not experience any nausea, vomiting, dyspepsia, or haematemesis during this time. Pain was mostly localised around the sternal notch, where she would have the sensation of a food bolus. Over the preceding two years, she had experienced about 7.5kg of weight loss with no constitutional symptoms of fevers or fatigue. Her past medical history included hypertension, dyslipidaemia, lower back pain, cold agglutinin disease, monoclonal B lymphocytosis, and peripheral arterial disease. 

On physical examination, she seemed largely in good health with no signs of malnourishment. Halitosis was not present, and neither were palpable lymph nodes in the axilla, neck, or supraclavicular region. Abdominal examination was normal with no pain localised to any region, specifically no epigastric tenderness or masses. 

**Figure 1:**
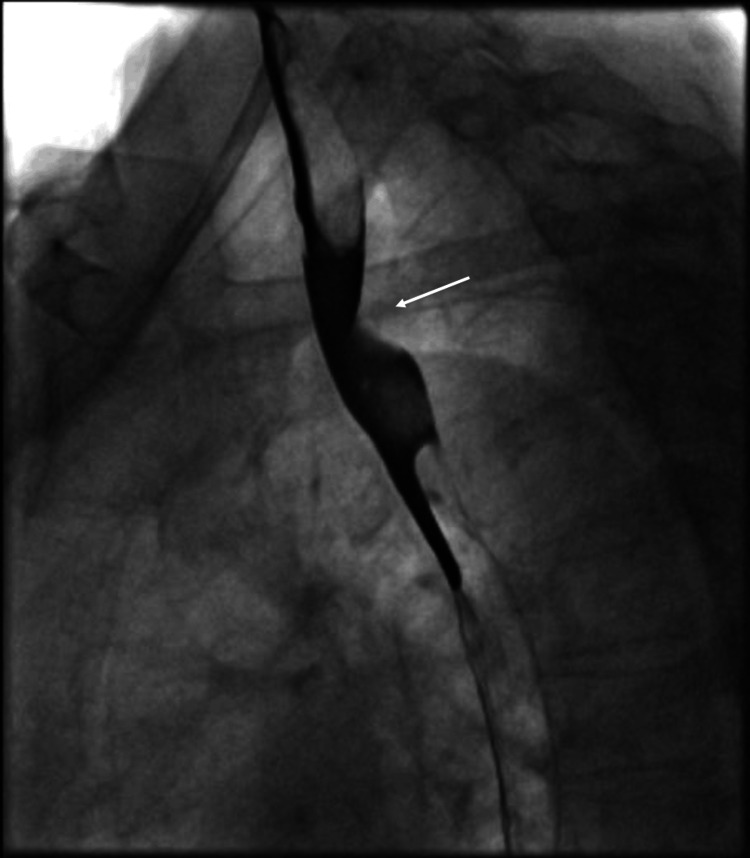
Barium swallow examination showing extrinsic compression of the proximal third of the oesophagus at the level of the aortic arch (white arrow)

**Figure 2:**
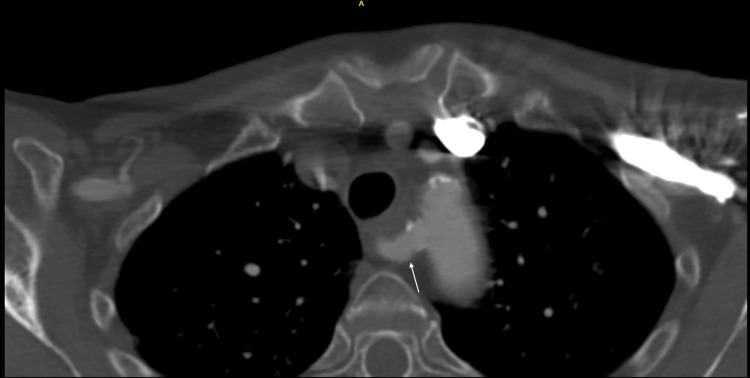
Axial view of CTA showing the posterior course of the retroesophageal ARSA (white arrow) CTA: Computed tomography angiography, ARSA: Aberrant right subclavian artery

**Figure 3:**
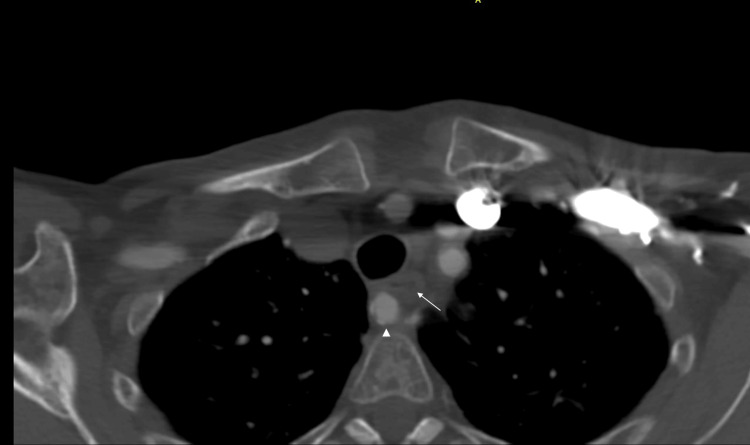
Axial view of CTA showing compression of oesophagus (white arrow) by the retroesophageal ARSA (white arrowhead) CTA: Computed tomography angiography, ARSA: Aberrant right subclavian artery

Given largely non-illuminating blood results, an oesophageal-gastroduodenoscopy was performed. However, no findings were noted, specifically no oesophageal webs, Zenker diverticulum, gastroparesis, trachealisation of the oesophagus, or corrosive pathology. Helicobacter pylori was also not isolated on histopathology. A barium swallow was then performed, which showed extensive external compression of the proximal third of the oesophagus at the level of the arch of the aorta, most indicative of dysphagia lusoria (Figure 1). Her subsequent computed tomography angiography (CTA) showed retroesophageal ARSA bifurcating from the ascending aorta independent of a brachiocephalic artery and causing external oesophageal compression (Figures 2-3).

**Figure 4:**
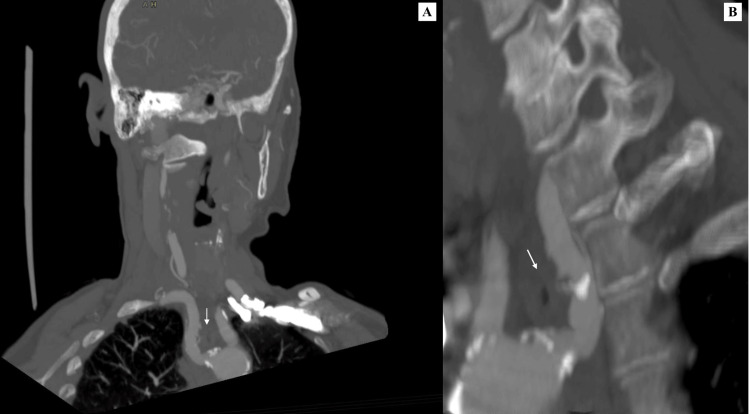
The CTA multi-planar reconstruction (MPR), both coronal (A) and sagittal (B) views, showing the posterior course of the right subclavian artery (white arrow), crossing the mediastinum and compressing the oesophagus. CTA: Computed tomography angiography

Given this definitive diagnosis, the patient was seen in the clinic where the management of dysphagia lusoria was explained. Despite ongoing symptoms, the patient decided to continue with conservative management and lifestyle modifications, which included eating smaller bites and increasing fluid intake. Although an extensive compression typically requires surgical intervention, our patient was stable at the two-year follow-up without the progression of the compression and further deterioration of the symptoms (Figure 4). 

## Discussion

Arteria lusoria is more common in females than males (55.3% vs. 44.7%), with the mean age of symptom onset being 49.9 ± 19.4 years (males 44.9 ± 18.1 years vs. females 54.0 ± 19.6 years, p = 0.0061) [1]. The majority of the patients with aberrant subclavian artery (ASA) are asymptomatic, and only around 5% to 10% will develop symptoms [1,2]. Patients are usually symptomatic at later stages due to the increment in the rigidity of the oesophagus and blood vessels, aortic elongation, and/or aneurysm formation [5]. 

A systematic review of 141 cases (15 cadaveric and 126 clinical cases) by Polguj et al. reported that more than two-thirds (71.2%) of the patients with arteria lusoria present with dysphagia, which is the most common symptom [1]. Dysphagia is followed by shortness of breath, chest pain, cough, and weight loss. Children can present with severe respiratory symptoms with an ASA. However, in adults, the trachea is more rigid, and dysphagia is the main complaint [2]. Klinkhamer noted the symptoms and signs of tracheoesophageal compression only in the case of ASA with truncus bicaroticus [2]. 

A CT could diagnose ASA with 100% sensitivity [6]. Giuliani et al. have recommended supra-aortic trunk imaging as a baseline investigation [7]. Recently, there has been emerging evidence of ARSA, especially in patients with genetic arteriopathies [7-9]. Scala et al. [8] reported a higher prevalence of the ARSA in Down syndrome fetuses compared to the euploid (23.6% (95% CI, 19.4-27.9%) vs. 1.02% (95% CI, 0.86-1.10%)), recommending ARSA as a clinical ultrasound marker for the prenatal diagnosis of Down syndrome. 

Although most ASA is silent, it can cause hemodynamic alterations in the cervicothoracic region, resulting in compressive and hemodynamic symptoms and necessitating surgical management [10]. However, surgical management is only indicated for symptomatic patients (recommendation class I, level C) or aberrant subclavian aneurysms ≥ 3 cm and KD ≥ 5.5 cm (recommendation class IIA, level C) given the risk of dissection and/or rupture in these patients [11]. Open, hybrid, and total endovascular approaches are the alternatives depending on the anatomical variations and hemodynamic alterations [12,13]. 

Konstantinou et al. conducted a systematic review of the database from 1990 to 2020 on the surgical outcome of 732 ASA patients by dividing patients into two groups: group A (453 patients) with open or hybrid surgery undergoing sternotomy/thoracotomy vs. group B (279 patients) with endovascular or hybrid surgery without open chest access [12]. This study showed that surgical intervention is associated with low mortalities (pooled early mortality rate: 1.62% in group A vs. 1.96% in group B) and higher symptom relief (99.52% in group A vs. 95.79% in group B).

A recent systematic review and meta-analysis by Loschi et al. also showed that open surgery, hybrid, and total endovascular repair are safer and more effective surgical procedures with satisfactory early and midterm outcomes [13]. However, given relatively newer and rapidly evolving vascular stent-graft technologies, it is too early to speculate about the long-term outcomes. 

In conclusion, ARSA without ectasia or aneurysmal degeneration in asymptomatic patients could be managed conservatively, especially in elderly patients. Dong et al. showed that the majority of the asymptomatic ARSA patients managed conservatively did not require surgical intervention [14]. However, although most studies recommend surgical intervention in symptomatic patients, our case describes an elderly female with arteria lusoria who, being symptomatic, was managed conservatively. Regular surveillance, preferably with a yearly CTA, is important to detect any change that necessitates future surgical intervention.

As there is a lack of consensus regarding ASA management, especially regarding symptomatic management, we believe that a large multicentre international registry-based prospective study is required in these patients to study the natural course of ASA, which can guide us to make definitive future recommendations. Therefore, it is important to involve all the stakeholders to make a precision-based, individualised management plan that best suits the patient. 

## Conclusions

Arteria lusoria, or ARSA, is an uncommon yet clinically important disease that can sometimes have devastating consequences. It could also pose life-threatening complications, like acute aortic syndrome, necessitating higher vigilance. However, there is still a lack of consensus on its diagnosis, follow-up, and treatment. Conservative treatment is a viable option, especially in elderly patients, which should be supported with regular surveillance to detect any complications that necessitate surgical intervention.
